# Collagen Crosslinking for Keratoconus

**Published:** 2011-07

**Authors:** Petrina Tan, Jodhbir S Mehta

**Affiliations:** 1 Department of Ophthalmology, Tan Tock Seng Hospital, Singapore; 2 Singapore Eye Research Institute, Singapore; 3 Yong Loo Lin School of Medicine, National University of Singapore, Singapore; 4 Singapore National Eye Centre, Singapore; 5 Duke-NUS Graduate Medical School, Singapore

In this issue of JOVR, Derakhshan and coauthors^1^ describe the results of crosslinking for patients with early keratoconus. Collagen crosslinking is a recently described technique of corneal tissue strengthening using riboflavin as a photosensitizer and ultraviolet A (UVA) radiation to promote the formation of intra- and interfibrillar covalent bonds by photosensitized oxidation.^2^ To date, it is the only published intervention that may retard the progression of keratoconus. Collagen crosslinking has also been sequentially combined with other treatment modalities, namely intrastromal ring segments and photorefractive keratectomy, for treatment of keratoconus.^3^ Other possible indications include keratectasia following laser in situ keratomileusis (LASIK)^4^, infectious keratitis and bullous keratopathy^5^.

In vitro studies on human and porcine corneas have shown a significant increase in corneal rigidity after crosslinking as indicated by an increase in Young’s modulus.^6^ This stiffening effect was found to be depth-dependent, i.e. anterior corneal layers were stiffer corresponding to maximum crosslinking effect. The first in vivo controlled clinical study was performed by Wollensak et al^7^ and included 23 eyes with moderate or advanced progressive keratoconus. This study showed that crosslinking was effective in halting the progression of keratoconus over a period of 4 years. The authors reported mean reduction of 2.01 D in maximum keratometry and 1.14 D in spherical equivalent. These initial results were later supported by other studies showing varying degrees of improvement in visual acuity and reduction in keratometry with a progressive trend in improvement depending on the duration of follow-up.^8^,^9^ Collagen crosslinking has also been found to be relatively safe. Wollensak et al^7^ reported no change in corneal and lens transparency, endothelial cell density, and intraocular pressure. Epithelial regeneration was completed normally within 4 days, and restoration of corneal sensitivity and repopulation of the corneal stroma occurred by 6 months.^10^ Few complications have been described including corneal scarring and stromal haze which require a variable duration for resolution^11^, and infectious keratitis including bacterial^12^, acanthamoeba^13^ and herpetic^14^ infections.

Derakhshan et al^1^ have published their observational study on the effect of crosslinking as primary treatment for patients with early keratoconus. Their study enrolled 22 patients and treated 31 eyes with mean follow-up of 6 months. Their results show significant improvement in uncorrected and best spectacle corrected visual acuity, and reduction in spherical equivalent and keratometric readings. These changes however, were less marked than previously published studies. Visual improvement in most patients began from the first month, slightly increased by the third month and remained stable until six months. No complications such as cataracts, raised intraocular pressure, or persistent epithelial defects were noted in this study. The effect of treatment appeared to be less as compared to previous studies which is likely due to a difference in treatment groups; previous studies enrolled patients with progressive keratoconus, while their study was performed on patients with early keratoconus. Even though the authors ascertained the penetration of riboflavin into the anterior chamber, the procedure was performed with the epithelium on.

This study offers some interesting insights into the use of collagen crosslinking for patients with early keratoconus. It is not unusual that the effect of the procedure was not as pronounced as in patients with moderate/progressive disease. Nevertheless, 77% of treated patients demonstrated an improvement in keratometry. Whether the latter translated into the possibility of subsequent optical correction with contact lenses was not addressed in the paper. However if this is actually the case, crosslinking may be advocated for patients with early disease. Longer term follow up is required to ensure that the observed changes are sustained. The unresolved issue is identifying the most suitable candidates for such treatment. For this purpose, analysis of early biomechanical changes may be a more sensitive means than keratometric or visual acuity changes.
